# High-grade Endometrial Carcinomas: Morphologic and Immunohistochemical Features, Diagnostic Challenges and Recommendations

**DOI:** 10.1097/PGP.0000000000000491

**Published:** 2018-12-14

**Authors:** Rajmohan Murali, Ben Davidson, Oluwole Fadare, Joseph A. Carlson, Christopher P. Crum, C. Blake Gilks, Julie A. Irving, Anais Malpica, Xavier Matias-Guiu, W. Glenn McCluggage, Khush Mittal, Esther Oliva, Vinita Parkash, Joanne K. L. Rutgers, Paul N. Staats, Colin J. R. Stewart, Carmen Tornos, Robert A. Soslow

**Affiliations:** Department of Pathology, Memorial Sloan Kettering Cancer Center (R.M., R.A.S.); New York University Medical Center and School of Medicine, Tisch Hospital (K.M.), New York; Department of Pathology, University Hospital, Stony Brook School of Medicine, Stony Brook (C.T.), New York; Department of Pathology, Oslo University Hospital, Norwegian Radium Hospital; Faculty of Medicine, Institute of Clinical Medicine, University of Oslo, Oslo, Norway (B.D.); Department of Pathology, University of California San Diego, San Diego (O.F.); Department of Pathology, Cedars-Sinai Medical Center, Los Angeles (J.K.L.R.), California; Department of Pathology, Karolinska Institutet, Stockholm, Sweden (J.A.C.); Department of Pathology, Brigham and Women’s Hospital, Harvard Medical School (C.P.C.); Department of Pathology, Massachussetts General Hospital, Harvard Medical School (E.O.), Boston, Massachussetts; Department of Pathology, University of British Columbia, Vancouver (C.B.G., J.A.I.); Department of Laboratory Medicine, Pathology and Medical Genetics, Royal Jubilee Hospital, Victoria (J.A.I.), BC, Canada; Department of Pathology, The University of Texas M. D. Anderson Cancer Center, Houston, Texas (A.M.); Department of Pathology, Hospital University Arnau de Vilanova; and Department of Pathology, Hospital University de Bellvitge, IRBLLEIDA, IDIBELL, University of Lleida, CIBERONC, Barcelona, Spain (X.M.-G.); Department of Pathology, Belfast Health and Social Care Trust, Belfast, UK (W.G.M.); Department of Pathology and Obstetrics and Gynecology, Yale School of Medicine and Yale School of Publich Health, New Haven, Connecticut (V.P.); Department of Pathology, University of Maryland School of Medicine, Baltimore, Maryland (P.N.S.); Department of Pathology, KEMH and School for Women’s and Infants’ Health, University of Western Australia, Perth, WA, Australia (C.J.R.S.)

**Keywords:** Carcinosarcoma, Clear cell carcinoma, Dedifferentiated carcinoma, Endometrioid carcinoma, Endometrium, FIGO Grade 3, High grade, Serous carcinoma, Undifferentiated carcinoma

## Abstract

This review of challenging diagnostic issues concerning high-grade endometrial carcinomas is derived from the authors’ review of the literature followed by discussions at the Endometrial Cancer Workshop sponsored by the International Society of Gynecological Pathologists in 2016. Recommendations presented are evidence-based, insofar as this is possible, given that the levels of evidence are weak or moderate due to small sample sizes and nonuniform diagnostic criteria used in many studies. High-grade endometrioid carcinomas include FIGO grade 3 endometrioid carcinomas, serous carcinomas, clear cell carcinomas, undifferentiated carcinomas, and carcinosarcomas. FIGO grade 3 endometrioid carcinoma is diagnosed when an endometrioid carcinoma exhibits >50% solid architecture (excluding squamous areas), or when an architecturally FIGO grade 2 endometrioid carcinoma exhibits marked cytologic atypia, provided that a glandular variant of serous carcinoma has been excluded. The most useful immunohistochemical studies to make the distinction between these 2 histotypes are p53, p16, DNA mismatch repair proteins, PTEN, and ARID1A. Endometrial clear cell carcinomas must display prototypical architectural and cytologic features for diagnosis. Immunohistochemical stains, including, Napsin A and p504s can be used as ancillary diagnostic tools; p53 expression is aberrant in a minority of clear cell carcinomas. Of note, clear cells are found in all types of high-grade endometrial carcinomas, leading to a tendency to overdiagnose clear cell carcinoma. Undifferentiated carcinoma (which when associated with a component of low-grade endometrioid carcinoma is termed “dedifferentiated carcinoma”) is composed of sheets of monotonous, typically dyscohesive cells, which can have a rhabdoid appearance; they often exhibit limited expression of cytokeratins and epithelial membrane antigen, are usually negative for PAX8 and hormone receptors, lack membranous e-cadherin and commonly demonstrate loss of expression of DNA mismatch repair proteins and SWI-SNF chromatin remodeling proteins. Carcinosarcomas must show unequivocal morphologic evidence of malignant epithelial and mesenchymal differentiation.

High-grade endometrial cancers include FIGO grade 3 endometrioid carcinomas, serous carcinomas, clear cell carcinomas, undifferentiated/dedifferentiated carcinomas, and carcinosarcomas. Typical examples of these histotypes are not difficult to diagnose based on careful examination of their morphologic features, allied with confirmatory immunohistochemistry if required. In some cases, the histopathologic and immunohistochemical characteristics are less clear-cut and overlap significantly, which makes accurate classification difficult. Even among specialist gynecologic pathologists, the interobserver reproducibility in the typing of high-grade endometrial carcinomas is suboptimal, with reported kappa values ranging between 0.55 and 0.68 1–5.

Some studies have shown prognostic differences between the histologic subtypes of high-grade endometrial cancers. In an analysis of 4180 cases by the Surveillance, Epidemiology, and End Results (SEER) Program, poorer outcomes were observed in patients with serous carcinoma and clear cell carcinoma compared with those with grade 3 endometrioid carcinoma 6,7. Other studies found that patients diagnosed with serous carcinoma had poorer survival than those with grade 3 endometrioid carcinoma 8,9. In contrast, 3 studies of high-grade endometrial carcinoma identified no significant differences in survival among patients with grade 3 endometrioid carcinoma, serous carcinoma, and clear cell carcinoma 10–12. Therefore, the issue of whether or not grade 3 endometrioid carcinoma is as clinically aggressive as serous carcinoma and clear cell carcinoma has not been conclusively settled. Much of this controversy is likely due to the suboptimal interobserver reproducibility among pathologists in the histotyping of high-grade endometrial carcinomas.

In this review, we present an overview of the histologic and immunohistochemical features of the different subtypes of high-grade endometrial carcinomas, including a discussion of the challenges in diagnosis and differential diagnosis. We present recommendations based on the available literature to assist pathologists in diagnosing these tumors.

## FIGO GRADE 3 ENDOMETRIOID CARCINOMA

### Definition

An endometrioid carcinoma with >50% solid architecture, or 6% to 50% solid architecture and diffuse marked nuclear atypia. The presence of oval or round glands, lined by columnar or cuboidal cells with low-grade oval or round nuclei which are typically pseudostratified, establishes endometrioid lineage. Squamous metaplasia (morular or nonmorular) is common and should not be included in the estimation of extent of solid architecture when grading these tumors.

### Key Morphologic Features

**FIG. 1:**
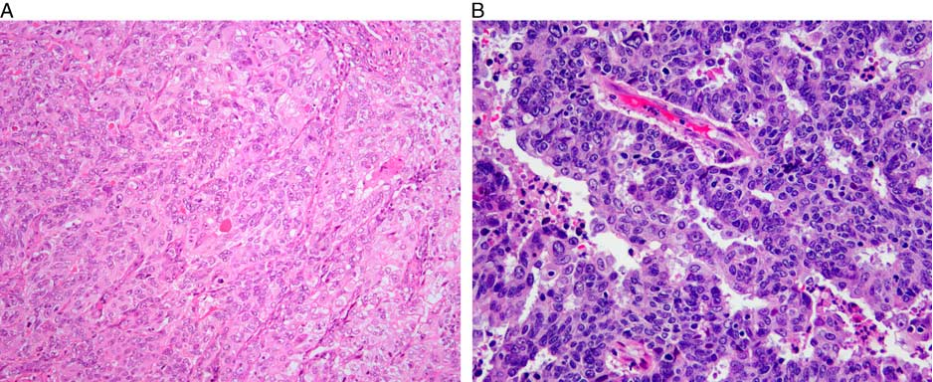
FIGO grade 3 endometrioid carcinoma. Solid architecture (A), glandular architecture with high nuclear grade (B).

Grade 3 endometrioid carcinomas frequently arise in association with endometrial hyperplasia. They are predominantly solid (Fig. 1A), but gland formation is usually seen at least focally (Fig. 1B), with evident transition from one component to the other. The solid component consists of large nests and occasional trabeculae. The cells in the solid component resemble those lining the glandular spaces. Nuclei usually have moderate (grade 2) atypia, and mucinous or squamous metaplasia is sometimes seen 13.

## ENDOMETRIAL SEROUS CARCINOMA

### Definition

**FIG. 2:**
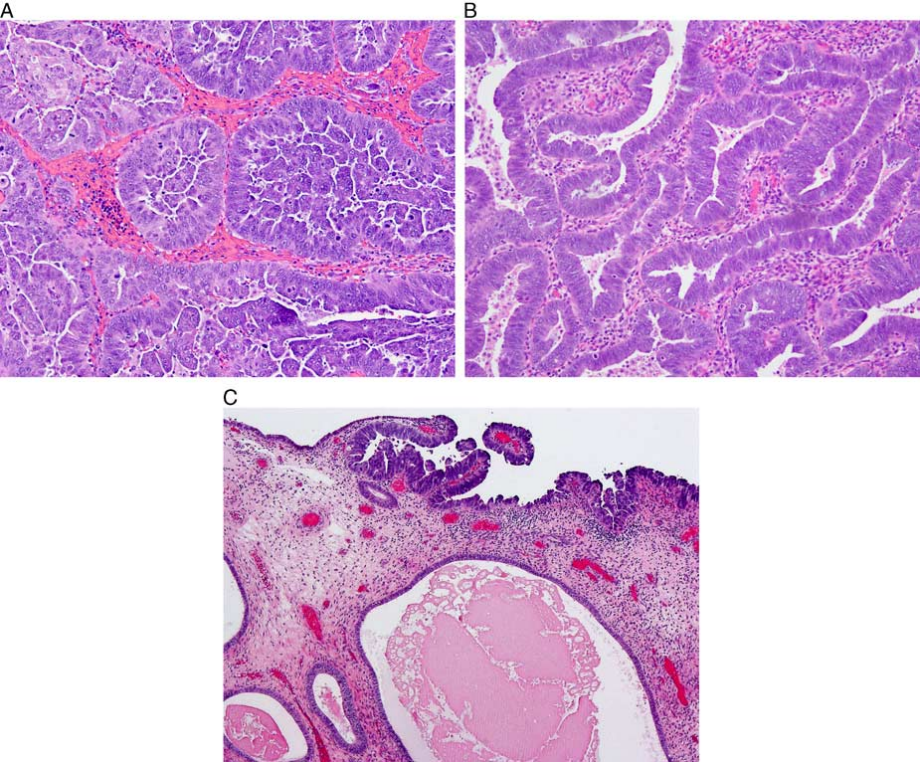
Serous carcinoma. Typical papillary and micropapillary architecture (A), glandular serous carcinoma recognized by highly atypical nuclei and high nuclear:cytoplasmic ratios (B), intraepithelial serous carcinoma involving atrophic endometrial polyp (C).

An endometrial carcinoma that usually shows marked and diffuse cytologic atypia and a papillary, glandular or solid architecture (Figs. 2A, B). Features definitional for endometrioid carcinoma and clear cell carcinoma (see below) are lacking. Nearly every case harbors a *TP53* mutation 14, which is associated with aberrant immunohistochemical expression of p53.

### Key Morphologic Features

Serous carcinoma generally develops in the background of atrophic endometrium, sometimes in a polyp (Fig. 2C). Most serous carcinomas show at least focal areas of papillary growth, sometimes with fibrovascular stalks (Fig. 2A); however, this finding may be absent. Budding and exfoliation of tumor cells are typically seen. The tumors may also exhibit irregular glands, often with slit-like spaces, but sometimes with round spaces, or a solid growth pattern (Fig. 2B). Psammoma bodies are found in one-third of cases. Nuclei are hyperchromatic, contain macronucleoli, are markedly atypical (grade 3), and pleomorphic/bizarre forms are often present. Numerous mitotic figures are usually found. Cytoplasm is often scant, but may be more abundant, with a clear or eosinophilic appearance 13,15,16. Some tumors lack marked cytologic atypia, but the tumor cells show hyperchromatic nuclei, increased nuclear:cytoplasmic ratios, numerous apoptotic bodies and frequent mitoses.

### Distinction of FIGO Grade 3 Endometrioid Carcinoma and Serous Carcinoma

#### Morphology

Recognition of key morphologic features detailed above will permit this distinction in most cases. Although serous carcinomas generally show greater degrees of nuclear atypia and polymorphism than grade 3 endometrioid carcinomas, they may both exhibit high-grade atypia and solid growth patterns, and serous carcinomas may show a predominantly glandular growth pattern 13,15,16. Grade 3 endometrioid carcinoma is typically predominantly solid, but glandular or less commonly papillary formation is seen at least focally, with transition from one component to the other 13.

**FIG. 3:**
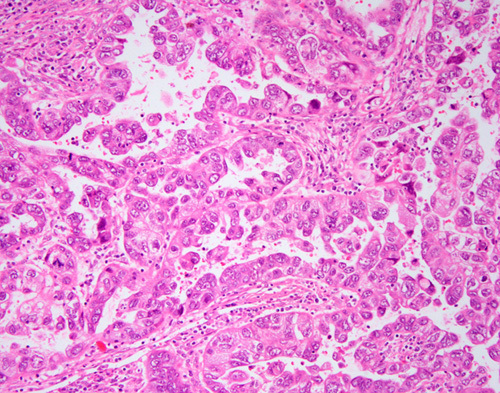
Diagnostically difficult endometrial carcinoma. This tumor presented in a 45-yr-old woman with atypical endometrial hyperplasia. Sequencing revealed a *POLE* mutation, but no *TP53* mutation. The final diagnosis was high-grade endometrioid carcinoma; the presence of a *POLE* mutation is prognostically favorable.

While the aforementioned features aid in distinguishing endometrioid carcinomas and serous carcinomas in many cases, there are some tumors that cannot be reproducibly classified (Fig. 3). In a study of 56 tumors diagnosed as high-grade endometrial carcinomas, 3 experienced gynecologic pathologists agreed on histotype in only 62.5% of cases, and there was disagreement with respect to the diagnosis of grade 3 endometrioid carcinoma versus serous carcinoma in 6 of 20 discrepant cases 2. In view of this difficulty, ancillary methods, such as immunohistochemistry and molecular testing, may be applied to aid classification.

#### Immunohistochemistry

When evaluating immunomarker studies, it is important to bear in mind that reported studies vary in the cut-off points used to assess positive and negative staining, making it difficult to compare the results of different studies.

In a study of 40 grade 3 endometrioid carcinomas and 24 serous carcinomas 17, estrogen receptor (ER), progesterone receptor (PR), p16, monoclonal carcinoembryonic antigen, and vimentin were expressed in 50% versus 54%, 42% versus 54%, 25% versus 92%, 3% versus 13%, and 81% versus 83% of tumors, respectively. This suggests limited discriminatory use for these markers; however, any degree of staining was scored as positive, probably masking the value of diffuse p16 expression in diagnosing serous carcinoma. That the extent of p16 expression might be important was illustrated by a study which found that serous carcinoma showed p16 expression in 90% to 100% of cells, compared with 10% to 90% of cells in grade 3 endometrioid carcinoma 18. Another study 2 using a panel of ER, PR, p16, p53, and PTEN found that the majority of serous carcinomas are negative for ER and PR, positive for PTEN, diffusely positive for p16 and exhibit aberrant mutation-type expression (diffusely and strongly positive or entirely negative) with p53, whereas grade 3 endometrioid carcinomas are more likely to be positive for ER and PR, negative for PTEN (correlating with genetic aberrations of *PTEN*^19^), focally positive for p16 and show wild-type staining for p53. However, exceptions to this typical staining pattern occur in both tumor types and in general a panel of markers is the most reliable approach 2.

The insulin-like growth factor II mRNA-binding protein family (IMP; IGFBP) consists of 3 proteins (IMP1, IMP2, and IMP3). IMP2 is expressed in virtually all serous carcinomas and grade 3 endometrioid carcinomas, but in one study, the former showed staining in >95% of tumor cells compared with ≤75% of tumor cells in the latter 20. IMP3 has been shown to be expressed in a majority (>90%) of serous carcinomas 21,22. A panel consisting of IMP3, PTEN, p53, and beta-catenin was applied to 103 endometrial cancers (including 31 serous carcinomas and 16 grade 3 endometrioid carcinomas). IMP3, PTEN, p53, and beta-catenin were detected in 17% versus 100%, 28% versus 90%, 56% versus 84%, and 28% versus 0% of grade 3 endometrioid carcinomas versus serous carcinomas, respectively 23. Another immunohistochemical analysis of 180 endometrial carcinomas including 34 grade 3 endometrioid carcinomas and 15 serous carcinomas 8 found that IMP3, ER, PR, PTEN, p53, and p16 were detected in 20%, 82%, 68%, 61%, 39%, and 19% of grade 3 endometrioid carcinomas and 63%, 50%, 46%, 100%, 69%, and 90% of serous carcinomas, respectively.

*ARID1A* is a tumor suppressor gene mutated in ∼50% of ovarian endometrioid and clear cell carcinomas, as well as a significant percentage of the corresponding uterine tumors, resulting in loss of immunoexpression of its protein product, BAF250a. Analysis of 190 high-grade endometrial cancers 24 showed loss of BAF250a expression in 46% of grade 3 endometrioid carcinomas compared with 9% of serous carcinomas. Aberrant p53 expression was found in 18% of grade 3 endometrioid carcinoma compared with 78% of serous carcinomas. In addition, loss of DNA mismatch repair (MMR) protein expression (MLH1, PMS2, MSH2, MSH6) was observed in 57% of grade 3 endometrioid carcinomas compared with 10% of serous carcinomas 24. These patterns of p53 and MMR protein expression have also been reported in other studies 25.

The expression of high-mobility group AT-hook 2 (HMGA2) was compared in 68 grade 3 endometrioid carcinomas and 71 serous carcinomas using tissue microarrays. Staining of any extent was present in 61% of serous carcinomas versus 25% of grade 3 endometrioid carcinomas. When present, staining was more diffuse in serous carcinoma. Serous endometrial intraepithelial carcinomas were also HMGA2-positive 26. In whole tissue sections, 91% of serous carcinomas were positive, usually with diffuse staining. All 5 cases of serous endometrial intraepithelial carcinoma were positive, as were 37% of endometrioid carcinomas, usually with focal staining 26.

WT1 expression is usually focally positive (in up to 30% of cases) or negative in uterine serous carcinoma 27, and this marker is therefore not routinely applied in the differential diagnosis between grade 3 endometrioid carcinoma and serous carcinoma at this anatomic site. However, since endometrioid carcinomas are usually WT1-negative, diffuse WT1 expression when present suggests a diagnosis of serous carcinoma, including derivation from an adnexal serous carcinoma.

In the most comprehensive study of potential markers useful in the distinction between grade 3 endometrioid carcinoma and serous carcinoma published to date, Han et al. 3 analyzed the diagnostic role of 12 proteins (ER, PR, p16, p53, Ki-67, PTEN, beta-catenin, vimentin, IMP3, TFF3, ARID1A, and HNF1B) in this differential diagnosis. TFF3 expression, ARID1A loss and beta-catenin expression had 100% specificity in diagnosing grade 3 endometrioid carcinoma, but relatively low sensitivity at 37%, 33%, and 7%, respectively. p53, p16, and IMP3 stained 94%, 80% and 63% of serous carcinomas, respectively, compared with 26%, 11%, and 11% of grade 3 endometrioid carcinomas 3.

**FIG. 4:**
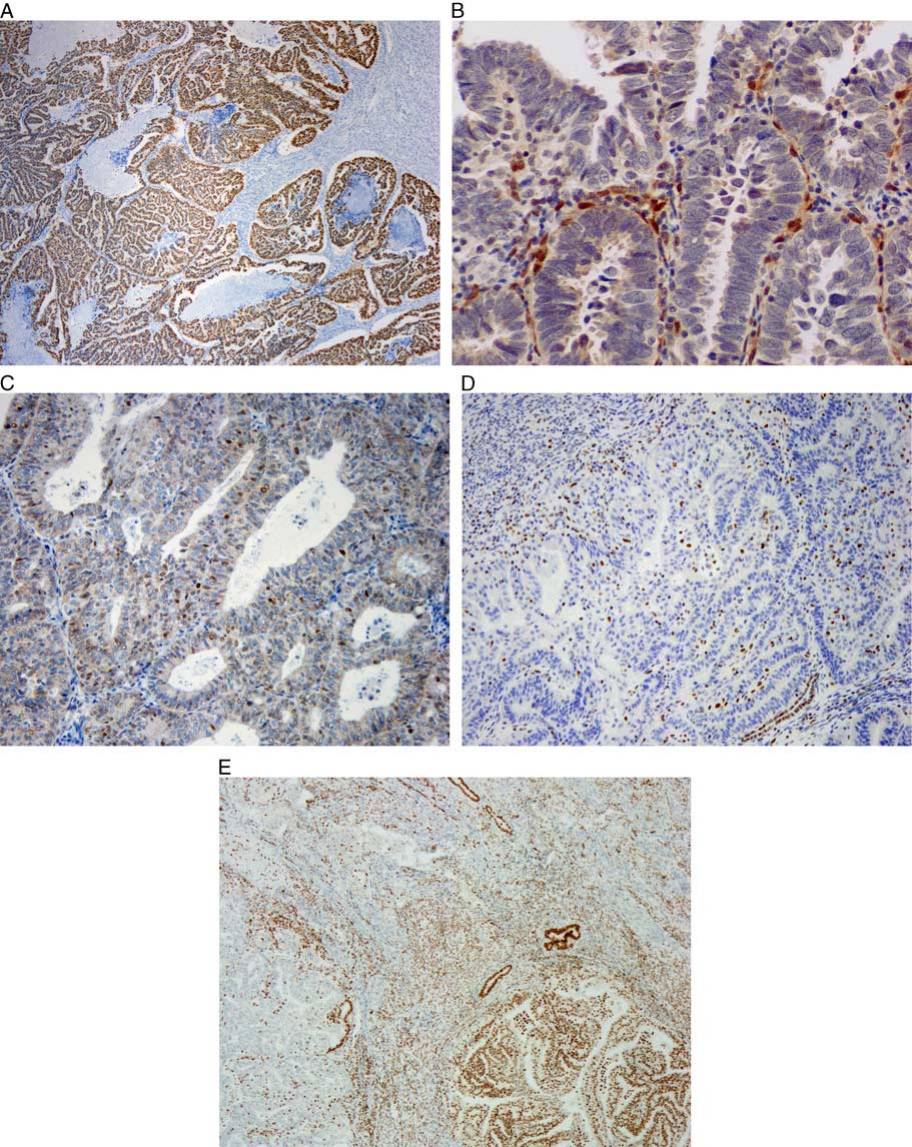
Immunohistochemistry useful in distinction of serous carcinoma and high-grade endometrioid carcinoma. (A) p53 overexpression (aberrant) may be seen in high-grade endometrioid carcinoma and serous carcinoma. (B) Null p53 phenotype (aberrant) may be seen in high-grade endometrioid carcinoma and serous carcinoma. Note positive internal control. (C) Wild-type p53 expression, not seen in serous carcinoma. (D) Loss of MLH1 expression (aberrant), not seen in serous carcinoma. (E) Geographic loss of ARID1A expression (left), not seen in most serous carcinomas.

In summary, although individual immunomarkers are differentially expressed in these tumors, no single marker is absolutely diagnostic of either grade 3 endometrioid carcinoma or serous carcinoma. Reaching an accurate, reproducible diagnosis appears to be feasible in most cases using a combination of careful morphologic assessment supplemented by the judicious use of immunohistochemistry with a panel of stains rather than a single marker (Box 1). In general, immunohistochemical markers that can be scored as “all-or-none” or at least as diffuse versus nondiffuse are likely to be assessed in a more reproducible manner than those that require estimation of the extent of staining or staining intensity. The former group includes p16, p53 (Figs. 4A–C), PTEN, DNA MMR proteins (Fig. 4D), and ARID1A (Fig. 4E). ER, despite its expression in a significant number of serous carcinomas, appears to be useful in combination with these markers, while the evidence for PR is less conclusive. The combination of ER, PR, p16, p53, vimentin, PTEN, and IMP3 was 100% concordant with morphology in the largest study to address this differential diagnosis, with the combination of ER, p16, and p53 being interpreted as the most informative when applying a limited panel of 3 markers 3; however, the role of IMP3 in diagnosing serous carcinoma is more difficult to assess given the different cut-offs applied in different studies. The presence of TFF3 staining, beta-catenin expression and loss of MMR protein expression appears useful in diagnosing grade 3 endometrioid carcinoma and these markers may be included in extended panels.

**BOX 1:**
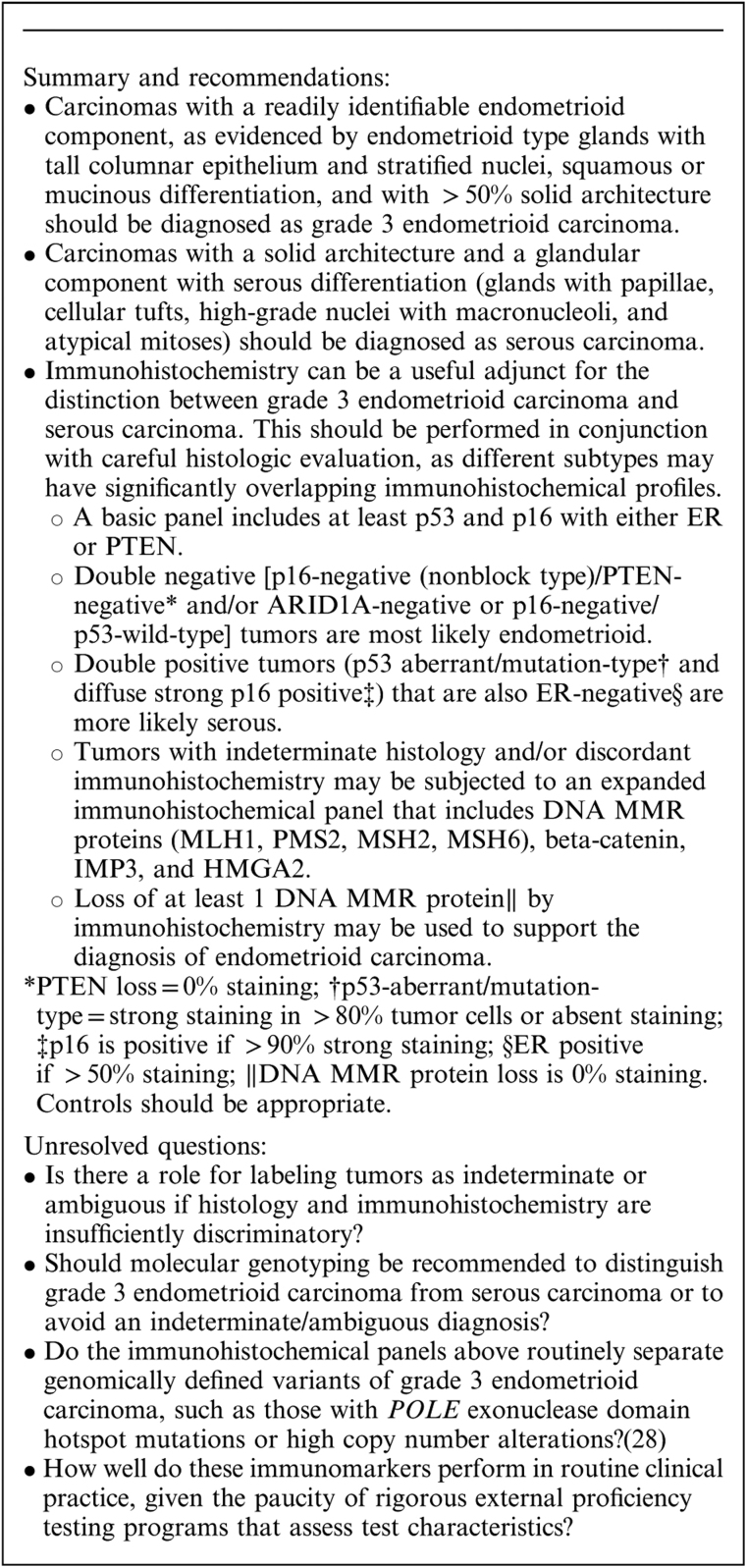
Distinction of FIGO Grade 3 Endometrioid Carcinoma and Serous Carcinoma

The role of HMGA2 in this differential diagnosis requires further research. Insufficient evidence is available to support the use of vimentin, Ki-67, HNF1B, WT1 and IMP2, and monoclonal carcinoembryonic antigen may be safely omitted from the antibody panel. Molecular classification of endometrial carcinomas has been shown to be superior to immunohistochemistry as an ancillary technique 29, but whether, and to what extent, it will replace immunohistochemistry remains to be seen, especially since it is more expensive, more time-consuming and not available in many pathology laboratories.

## CLEAR CELL CARCINOMA

### Definition

**FIG. 5:**
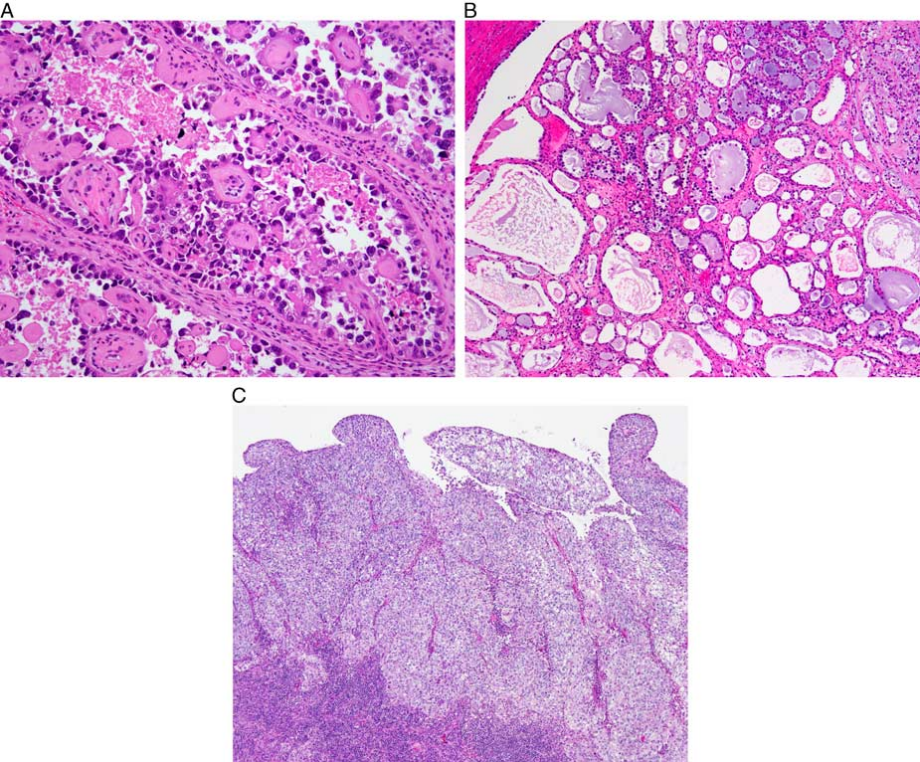
Clear cell carcinoma. Papillary architecture (A), tubulocystic architecture (B), solid architecture (C).

An endometrial carcinoma demonstrating a combination of papillary (small round papillae lacking overt stratification; Fig. 5A), tubulocystic (Fig. 5B) and/or solid (Fig. 5C) architectural patterns, with cuboidal or polygonal cells containing nuclei with a variable degree of pleomorphism (although usually lacking overt pleomorphism). Hobnail tumor cells and cytoplasmic clearing are often present but are not required for diagnosis.

### Key Morphologic Features

Clear cell carcinomas of the endometrium are uncommon neoplasms that are likely to be overdiagnosed. Accuracy in the diagnosis of clear cell carcinoma is best achieved by strict adherence to morphologic criteria that are essentially based on the prototypical profile of analogous tumors of the ovary, where clear cell carcinomas are more common and are more reproducibly diagnosed 30–32. The validity of such an extrapolation is supported by the fact that ovarian and endometrial clear cell carcinomas have been shown to have highly similar gene expression, proteomic, morphologic, and immunohistochemical profiles 33–37.

#### Architectural Patterns

Clear cell carcinoma should display at least one of 3 architectural patterns: solid, papillary, and/or tubulocystic. More than 80% of cases show an admixture of 2 or more patterns 34,38. The tubulocystic pattern is the most commonly encountered, and is at least focally present in most cases 4,34,38, but the papillary pattern is most frequently (28%–41%) predominant 34,38–41.

*Papillary pattern* (Fig. 5A). The papillae of clear cell carcinoma are most commonly in the form of small rounded papillae. Stromal hyalinization is present in the majority of tumors but typically involves only a proportion of the papillae 34. Other papillary patterns include architecturally complex papillae with hierarchical branching, micropapillae, long and slender papillae, or other nonspecific papillary formations 34,40. The papillae of clear cell carcinoma are lined by hobnail, cuboidal, or polygonal cells with clear or eosinophilic cytoplasm, and should not show extensive nuclear stratification, cellular tufting, or detached cellular budding.

*Tubulocystic pattern* (Fig. 5B). The tubulocystic pattern represents a morphologic spectrum from glands/tubules to cystic formations 34,38–43. At the tubular/glandular end of this spectrum, the glands are relatively uniform and display rounded contours with open lumina. They may be extensively confluent and “back-to-back” or show an abundance of interglandular stroma. Increasing cystic dilatation of the glands is usually accompanied by less confluence, although fully cystic units may also be entirely confluent. Tubular glands are lined by polygonal, low cuboidal cells with clear to eosinophilic cytoplasm or by hobnail cells. Cystic glands may be lined by similar cells or by a bland, flat cellular population. The interglandular stroma may be hyalinized, myxoid, inflamed, edematous, or fibroblastic. Extensive nuclear stratification, cellular tufting, or detached cellular budding should not be seen.

*Solid pattern* (Fig. 5C). The solid pattern is almost invariably admixed with other patterns and is characterized by diffuse sheets of polygonal cells with well-defined cell borders, with interspersed thin fibrous septa 34,38–43. Clear cells usually predominate in the solid areas, although a conspicuous population of oxyphilic cells is not infrequent 34.

#### Cytologic Features

*Cell types and stratification*. The cell types that may be seen in clear cell carcinoma include polygonal cells with clear or eosinophilic cytoplasm and well-defined cell membranes, hobnail cells, and attenuated hobnail cells (flat cells) 34,41. Nuclear stratification in the epithelium lining glands and papillae may be focally present and is usually not prominent 34. Cellular tufting or detached cellular budding should not be diffusely present in clear cell carcinoma 34, and squamous differentiation should be absent.

*Atypia*. A given case may display wide variation in the degree of cytologic atypia but many cases show a background of relative monomorphism, with scattered or clustered cells exhibiting larger, pleomorphic nuclei 34. Diffuse severe nuclear atypia is not characteristic of clear cell carcinoma.

*Mitotic index*. The mitotic index may be quite variable between tumors and even within the same tumor, but overall, it is generally low 4,34,41. In one study of 21 cases 41, 52% had 0 to 1 mitotic figures per 10 high power fields, 29% showed 2 to 5 mitotic figures per 10 high-power fields and 19% had ≥6 mitotic figures per 10 high-power fields. Putative cases of clear cell carcinoma exhibiting very high levels of mitotic activity, especially when accompanied by severe atypia, warrant careful examination for other characteristic features of clear cell carcinoma.

### Immunohistochemical Features

The diagnosis of clear cell carcinoma should primarily be based on its distinctive morphologic features described above. However, immunohistochemistry can be useful in some specific scenarios: (1) ascertaining whether focal areas of clear cells in an endometrial carcinoma represent true clear cell carcinoma; and (2) evaluation of a tumor which shows some morphologic features that are suggestive but not diagnostic of clear cell carcinoma in a limited sample (eg, biopsy or curettage).

**FIG. 6:**
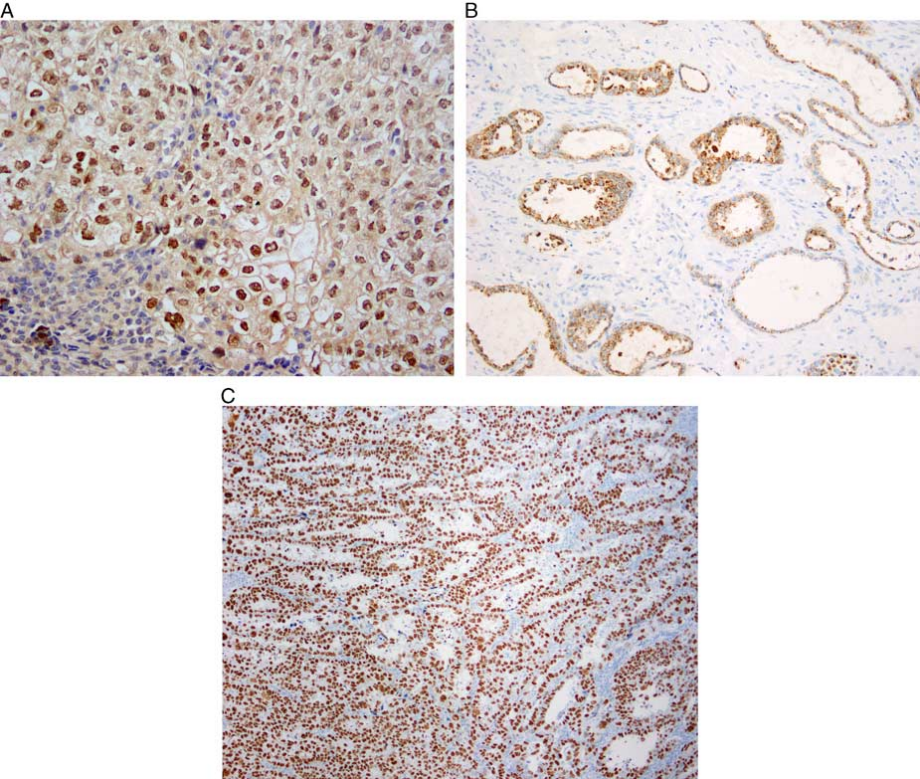
Immunohistochemistry in clear cell carcinoma. Hepatocyte nuclear factor 1-beta (HNF1B) is frequently expressed in clear cell carcinoma (A), as is Napsin A (B). A significant minority of endometrial clear cell carcinomas can display aberrant p53 staining (C).

The typical immunohistochemical profile of clear cell carcinoma is HNF1B-positive (Fig. 6A), Napsin A-positive (Fig. 6B), ER-negative and PR-negative and p53-wild-type 3,4,17,44–55. This immunohistochemical panel is more useful in the distinction of clear cell carcinoma from endometrioid carcinoma than from serous carcinoma. The extent of expression of Napsin A and HNF1B in serous carcinomas and endometrioid carcinomas is significantly lower than that in clear cell carcinomas. HNF1B has high sensitivity for endometrial clear cell carcinoma, but its specificity is lower than in ovarian clear cell carcinoma. Moreover, there are different antibodies against HNF1B, which vary in quality. Use of monoclonal Napsin A is recommended, as the polyclonal antibody lacks specificity 56. A small percentage of prototypical endometrial clear cell carcinomas are ER-positive, and clear cell change in endometrioid carcinoma may be accompanied by significant diminution of ER and PR expression 52; therefore ER and PR should always be used in conjunction with a panel of other markers 57.

Aberrant mutation-type p53 immunohistochemical expression is seen in up to one third of otherwise typical clear cell carcinomas 44,47,58,59 (Fig. 6C) and these cases are morphologically indistinguishable from p53-wild-type cases 47,58. Nevertheless, p53 immunohistochemistry can still provide useful information, as wild-type p53 staining minimizes the probability that the tumor is a true serous carcinoma 5, and mutation-type p53 expression is an adverse prognostic factor in histotypically ambiguous tumors 60. There is some evidence that p53-aberrant clear cell carcinomas show a more aggressive, “serous-like,” peritoneal pattern of spread 58,59. At the molecular level, ∼14% of morphologically and immunophenotypically characteristic clear cell carcinomas display a profile of mutations (mutations in *TP53* and *PPP2R1A*; wild-type *PTEN*, *CTNNB1*, and *ARID1A*) typically seen in serous carcinoma 47,48. These findings suggest that a subset of tumors diagnosed as clear cell carcinomas might represent manifestations of extreme morphologic mimicry by serous carcinomas 48,61.

The serous carcinoma-associated markers p16 and IMP3 may be expressed in clear cell carcinoma, and are not particularly useful in the distinction between these 2 tumor types 17,18,21,22,62,63. Although IMP2 and HMGA2 are frequently expressed in serous carcinomas 20,26, there are insufficient data about their expression in clear cell carcinoma. Similarly, the diagnostic value of AMACR (α-methylacyl-coenzyme-A racemase or p504s), which has been reported to be frequently positive in clear cell carcinoma, requires further study 64,65. Although loss of DNA MMR protein expression is not useful in the distinction of clear cell carcinoma and endometrioid carcinoma, this finding would favor clear cell carcinoma over serous carcinoma 24,44,66. In 2 recent studies, MMR deficiency was observed in 19% of clear cell carcinomas 67 and 0% of serous carcinomas 68.

### Differential Diagnosis of Clear Cell Carcinoma

Prototypical examples of endometrial clear cell carcinoma are characterized by a distinctive set of histopathologic features (described above) that enables their distinction from other histotypes 6,7,10,11,34,38–43,69–100. The significant interobserver variation that has historically been associated with the diagnosis of endometrial clear cell carcinoma is in large part related to the propensity for some high-grade (or less commonly low-grade) endometrial carcinomas of other types to contain clear cells and overlap with clear cell carcinoma. In such cases, definitive distinction of clear cell carcinoma from endometrioid carcinoma or serous carcinoma, or a mixed carcinoma with a clear cell component, may be challenging; this is compounded by the relative rarity of endometrial clear cell carcinoma.

#### Distinction of Clear Cell Carcinoma and Endometrioid Carcinoma

Since clear cell carcinoma is classified and managed as a high-grade carcinoma, it is important to distinguish it from low-grade endometrioid carcinoma, with which it may display morphologic overlap 82,90. It is not clear whether the prognosis for patients with clear cell carcinoma differs from that of patients with grade 3 endometrioid carcinoma (or serous carcinoma), with comparably sized bodies of published literature in support of the affirmative 6,7,40,69,70,72,81,87,88,97 and of the negative 10,11,71,73,80,87,91,94,96. Nevertheless, it is important to distinguish clear cell carcinoma from other histotypes, because: (a) inaccurate histotyping may obscure potentially significant differences between histotypes, including stage distribution, patterns of tumor spread and patterns of recurrence 11,39,89,93; (b) there is some evidence that patients with clear cell carcinoma are at increased risk for venous thromboembolic events 78,85,95; (c) in some institutions, there is a higher likelihood that adjuvant therapy would be recommended for a patient with stage I clear cell carcinoma than for a stage I grade 3 endometrioid carcinoma 101; (d) strict adherence to diagnostic criteria by pathologists will aid study of the molecular profile of clear cell carcinoma 16,102.

**FIG. 7:**
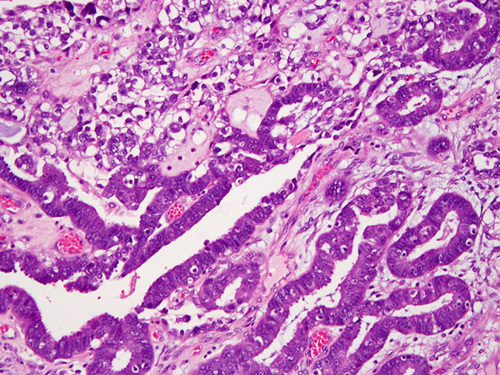
Mixed clear cell and endometrioid carcinoma. Note stark difference in cell shape, nuclear features and growth patterns.

Between 12% and 30% of clear cell carcinomas reportedly contain an endometrioid component 38–41,83,89,100. The proportion of these tumors that represent true mixed carcinomas 103 (Fig. 7) rather than a single histotype displaying phenotypic diversity 61 is unclear and the wide range in incidence is likely indicative of interobserver variability in the classification of these tumors 1,4. It is likely that true mixed endometrioid and clear cell carcinoma is very uncommon. Areas of morphologic overlap between clear cell carcinoma and endometrioid carcinoma are largely attributable to the presence of clear cells in endometrioid carcinomas, which may occur for several reasons:

- Endometrioid carcinoma with secretory change: secretory change in endometrioid carcinoma is characterized by subnuclear and/or supranuclear vacuoles of glycogen in the epithelial cells of an otherwise typical endometrioid carcinoma 104,105. The cells are columnar 38, compared with the polygonal cells of most clear cell carcinomas. Clear cell carcinomas generally show at least focal higher nuclear grade than endometrioid carcinomas with secretory change 38. These differences are less useful in high-grade endometrioid carcinoma.
- Endometrioid carcinoma with clear cell squamous differentiation: squamous differentiation in endometrioid carcinoma may have a variety of appearances, including rounded morules, spindled formations, plaque-like growth, pseudopapillae, micropapillae, and individual keratinized cells 106. All these patterns may display prominent cytoplasmic clearing, usually but not always due to glycogenation, that can mimic clear cell carcinoma 107. Avoiding mischaracterization of squamous differentiation as clear cell carcinoma requires recognition of the morphologic spectrum of squamous differentiation in endometrioid carcinoma, the finding of clear cell areas contiguous with more typical squamous differentiation or endometrioid glands, and the absence of cytoarchitectural patterns of clear cell carcinoma.
- Endometrioid carcinoma with nonspecific clear cell change: some endometrioid carcinomas of nonsecretory type exhibit nonspecific clear cell change within the glandular elements 106. Features useful in the distinction from clear cell carcinoma include the absence of the typical architectural patterns of clear cell carcinoma, transitions to typical glandular endometrioid carcinoma and foci of squamous differentiation.

Immunohistochemistry may be of some value in such cases but there can be significant immunophenotypic overlap and markers are often not particularly useful. Negative staining with ER and PR would favor a diagnosis of clear cell carcinoma but these markers can be negative in clear cell areas in endometrioid carcinomas, especially when they exhibit squamous differentiation. While HNF1B and Napsin A are useful markers of clear cell carcinoma, they (especially HNF1B) may also be expressed in clear cells in endometrioid carcinomas.

### Distinction of Clear Cell Carcinoma and Serous Carcinoma, and Mixed Clear Cell/Serous Carcinoma

Although prototypical examples of serous and clear cell carcinoma are readily distinguished, some cases present diagnostic problems 1,2,4. This is due to a number of factors such as the presence of clear cells in some serous carcinomas and serous-like features in some clear cell carcinomas. Current clinical management of patients with serous carcinoma, clear cell carcinoma and mixed serous carcinoma/clear cell carcinoma is not substantially different 101. However, this may change in the future, and accurate diagnosis will facilitate meaningful study of the clinicopathologic and genomic profiles of each histotype. Immunohistochemical markers useful in the distinction between serous and clear cell carcinoma have been discussed previously.

#### True Mixed Serous-Clear Cell Carcinomas

**FIG. 8:**
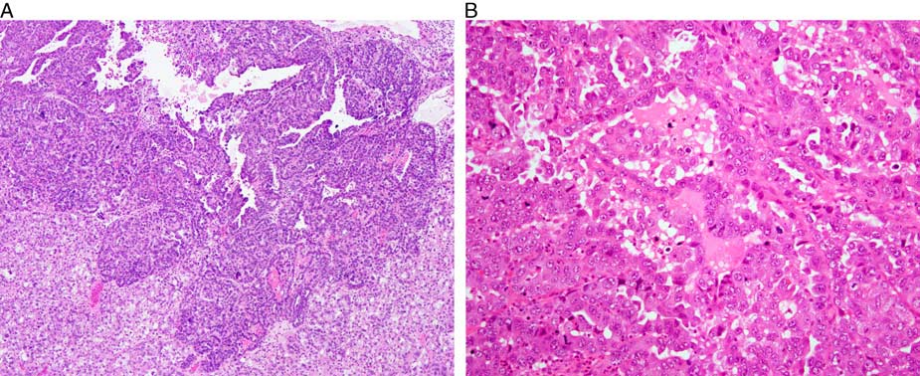
Clear cell carcinoma versus serous carcinoma. (A) Mixed serous and clear cell carcinoma. Serous carcinoma with overtly pleomorphic nuclei, stratification, tumor cell budding and slit-like spaces is found at the top of the image. (B) High-grade endometrial carcinoma displaying morphologic features overlapping those of clear cell carcinoma and serous carcinoma. The high mitotic index and nuclear pleomorphism are most characteristic of serous carcinoma.

A mixed endometrial carcinoma is defined in the latest World Health Organization (WHO) classification as a carcinoma composed of 2 or more histologic subtypes, in which the minor component constitutes 5% or more of the tumor, and in which the 2 components are recognizable on hematoxylin/eosin–stained sections 103. Each of the components must be spatially distinct from the other(s), and each must exhibit morphologic and immunophenotypic features that, viewed in isolation, is fully diagnostic of one histotype 61. Since evidence from molecular studies suggests that there is a high degree of morphologic mimicry in mixed carcinomas, it is imperative that each component is morphologically and immunophenotypically prototypical 61. As defined, true mixed serous-clear cell endometrial carcinomas are extremely uncommon (Fig. 8A).

#### Serous Carcinomas With Cytoplasmic Clearing

Serous carcinomas that contain cells with clear cytoplasm are much more likely to represent pure serous carcinomas than mixed serous-clear cell carcinomas. The evidence in favor of this interpretation includes: (1) the frequent presence of “clear cells” in endometrial carcinomas of various histotypes minimizes the importance of cytoplasmic clearing as a stand-alone indicator for clear cell carcinoma. (2) Data from ovarian carcinomas, in which histotyping is more reproducible 30–32, indicate that serous carcinomas with clear cells have a morphologic, immunophenotypic and molecular profile that is more consistent with serous carcinoma than clear cell carcinoma 108–110. (3) One seminal study found that in serous carcinomas with clear cells, none of the cases had a tubulocystic pattern, and serous endometrial intraepithelial carcinoma was present in a high proportion of the cases 111.

#### Clear Cell Carcinoma With Features that Overlap With Serous Carcinoma (Histologically Ambiguous Carcinomas)

These are the most challenging tumors to classify. The existence of truly ambiguous tumors in the clear cell carcinoma/serous carcinoma spectrum is one likely contributing factor to the interobserver variability that exists in the histotyping of high-grade endometrial carcinomas 1,2,4. These tumors have hybrid morphologic features overlapping those of serous carcinoma and clear cell carcinoma (Fig. 8B). The frequency of aberrant mutation-type p53 staining in these cases (36%) 112 is comparable with that reported in conventional clear cell carcinoma (33%–38%) 44,47,58,59, and substantially less than that expected in serous carcinoma. Immunohistochemistry, in our experience, has not proven to be very useful in the categorization of this group of cases. The true nature of these cases is unclear, and since they are not prototypical clear cell carcinoma or serous carcinoma, we recommend their provisional categorization in the “ambiguous” category, with descriptive diagnoses such as “High-grade carcinoma with clear cell and serous features” until they can be better categorized by novel modalities in the future. However, such a diagnosis should be made sparingly and only in those cases that defy classification after thorough morphologic and immunohistochemical evaluation.

In summary, otherwise typical serous carcinomas with clear cells should be categorized as serous carcinomas, and serous carcinomas with spatially distinct areas of clear cell carcinoma should be categorized as mixed serous carcinoma-clear cell carcinomas. Cases that are not morphologically typical of either histotype should be reported descriptively (Box 2).

**BOX 2:**
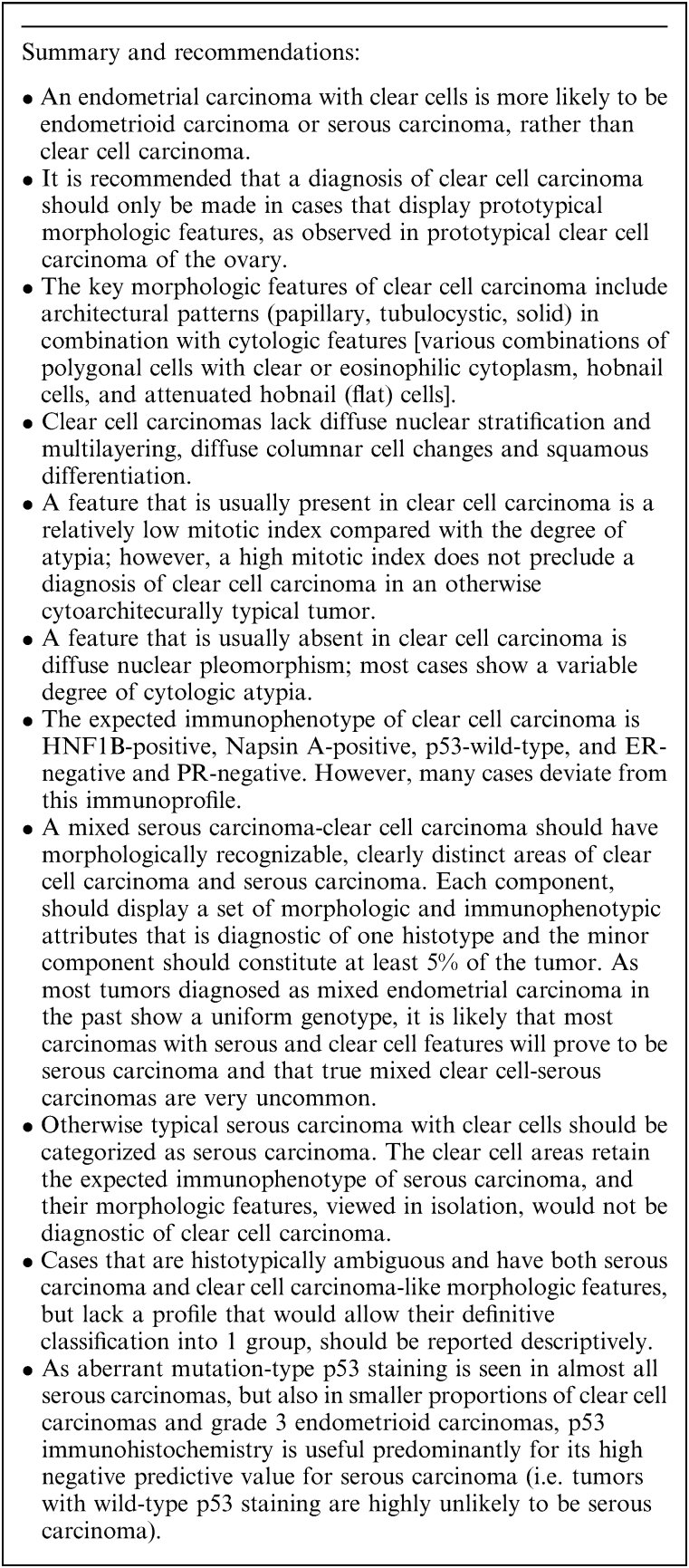
Clear Cell Carcinoma

## UNDIFFERENTIATED CARCINOMA AND DEDIFFERENTIATED CARCINOMA

### Definition

Undifferentiated carcinoma is a solid-pattern tumor lacking overt morphologic evidence of epithelial differentiation, except by immunohistochemistry, where focal (or rarely diffuse) cytokeratin and epithelial membrane antigen (EMA) expression is usually found. Dedifferentiated carcinoma is an undifferentiated carcinoma found in combination with an endometrioid carcinoma that is typically low-grade.

The 2003 WHO classification defined undifferentiated carcinoma simply as lacking any evidence of differentiation 113. However, reproducible recognition of undifferentiated carcinoma was hampered by the lack of a proper operational definition until the 2014 WHO classification 114, in which undifferentiated carcinoma was described as a monomorphic neoplasm which may resemble lymphoma, plasmacytoma, high-grade endometrial stromal sarcoma or small cell carcinoma 115. Undifferentiated carcinoma and dedifferentiated carcinoma of the endometrium should be considered specific entities and these diagnoses should not be used for histologically and/or immunophenotypically ambiguous high-grade tumors that are difficult to classify 116.

Undifferentiated and dedifferentiated carcinomas are clinically aggressive malignancies that are probably under-recognized 115,117,118. They have been widely recognized only in the past decade, and data pertaining to them is based upon a limited number of relatively small studies 115,117–126. In our experience, undifferentiated carcinomas and dedifferentiated carcinomas together represent ∼10% of high-grade endometrial carcinomas and hence about 2% of endometrial carcinomas overall 127. Silva et al. 124 reported that undifferentiated carcinomas accounted for 9% of all endometrial carcinomas; however, this report was from a major tertiary cancer center and it is unclear whether the frequency was inflated due to inclusion of referral and consultation cases.

### Key Morphologic Features

**FIG. 9:**
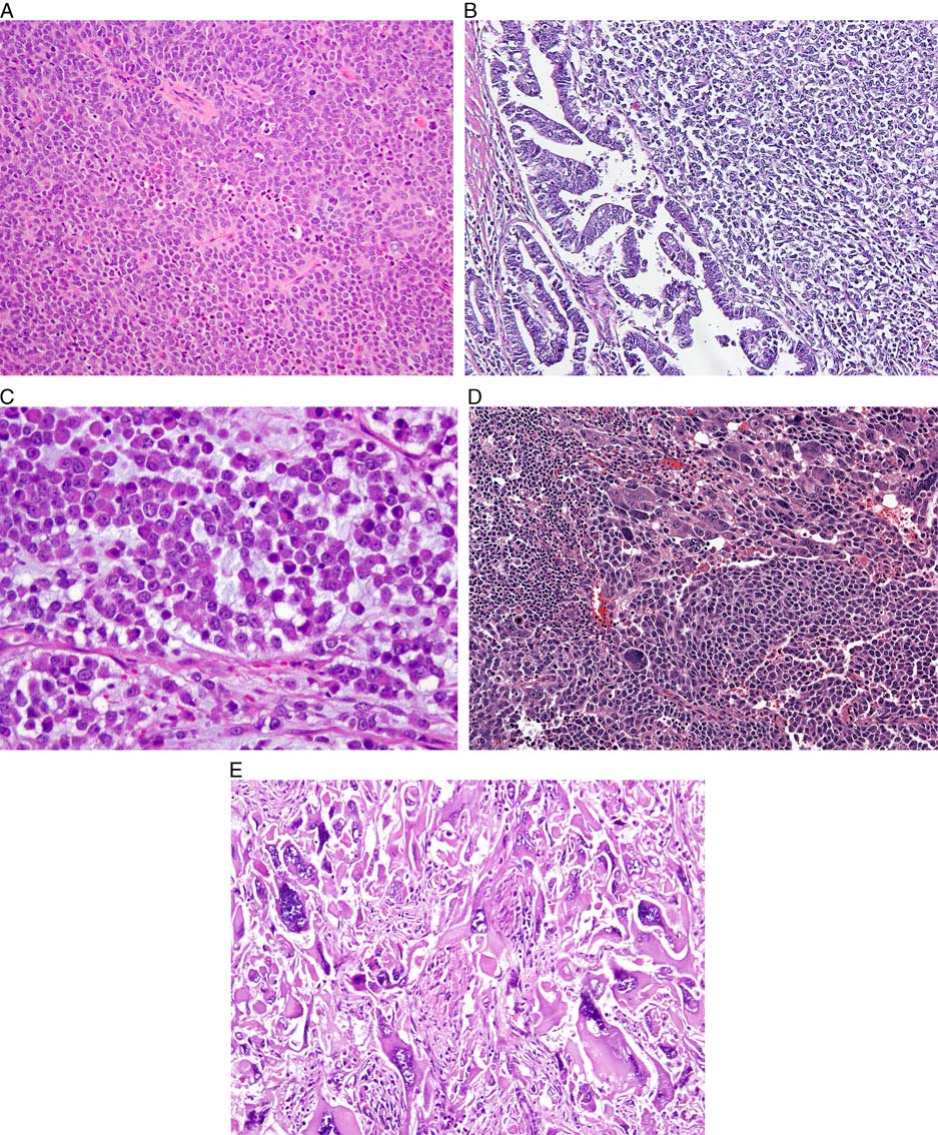
Undifferentiated and dedifferentiated carcinoma. (A) Prototypical undifferentiated carcinoma. (B) Dedifferentiated carcinoma. (C) Undifferentiated carcinoma containing rhabdoid cells in a myxoid matrix. (D) Undifferentiated carcinoma containing monomorphic and pleomorphic cells. (E) Undifferentiated carcinoma containing multinucleate giant cells. This pattern corresponds to the “giant cell carcinoma” of Scully and is unrelated to other types of undifferentiated carcinomas discussed herein.

Undifferentiated carcinoma is a monomorphic neoplasm composed of small to intermediate-sized cells arranged in sheets without any obvious glandular differentiation. They frequently exhibit a characteristically “dyscohesive” pattern and the low-power appearance raises a differential diagnosis of lymphoma, plasmacytoma, high-grade endometrial stromal sarcoma or small cell carcinoma (Fig. 9A) 115,118,121,124.

Approximately 40% of undifferentiated carcinomas are associated with a component of FIGO grade 1 or 2 endometrioid carcinoma; these cases are termed “dedifferentiated carcinoma” (Fig. 9B). It is possible that in some cases of undifferentiated carcinoma, an antecedent low-grade component was overgrown by the undifferentiated element. When present, the differentiated low-grade endometrioid component is typically found lining the endometrial cavity, with the undifferentiated component present deep to it. This may account for a diagnosis of low-grade endometrioid carcinoma in a biopsy specimen and dedifferentiated carcinoma at hysterectomy. In rare cases, low-grade endometrioid carcinoma is present in the uterus and the undifferentiated component is only seen in the metastases, which may be identified synchronously or subsequently 117,118,121,124. Rare cases contain foci of FIGO grade 3 endometrioid carcinoma juxtaposed with undifferentiated carcinoma 118.

Additional morphologic features that are present in some undifferentiated and dedifferentiated carcinomas include focal alveolar, nested and vaguely corded or trabecular growth patterns, rhabdoid/plasmacytoid morphology (vesicular nucleus containing a large nucleolus and abundant pink cytoplasm; Fig. 9C), focal marked nuclear pleomorphism (Fig. 9D), multinucleation, spindling, and “abrupt” keratinization 118,124,128,129. Some tumors also show prominent tumor-infiltrating lymphocytes and myxoid stroma. The morphologic criteria have continued to evolve and vary to some extent between studies. For example, the degree of permissible neuroendocrine differentiation (as judged immunohistochemically) was initially restricted to <10% of tumor cells 115, but in one subsequent study of undifferentiated carcinoma, “diffuse staining” was recorded in 9% of cases; we recommend that neuroendocrine marker positivity in undifferentiated carcinoma should be limited to <10% of the tumor cells 129.

### Immunohistochemical Features and Genotype

**FIG. 10:**
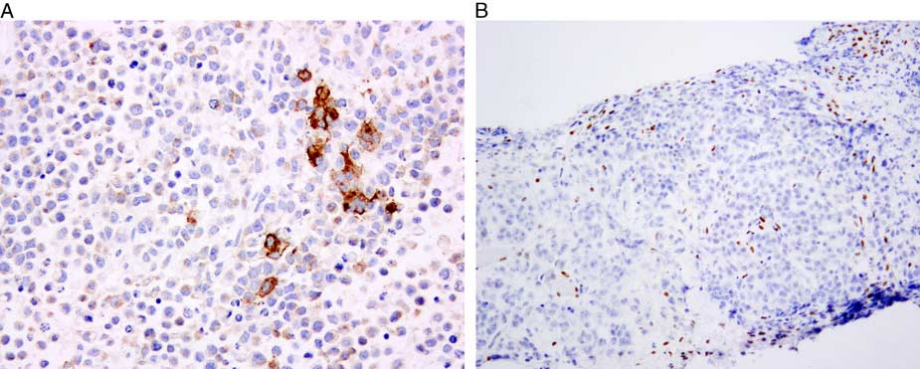
Immunohistochemistry in undifferentiated carcinoma of monomorphic type. (A) Focally intense labeling with CK18 in the absence of PAX 8 expression (latter not shown). (B) Loss of BRG-1 (SMARCA4) expression.

The majority of undifferentiated carcinomas (and the undifferentiated component of dedifferentiated carcinoma) lack expression of PAX8, ER, and PR but up to 20% of tumors may show focal staining with these markers 121,130. P53 expression is usually wild-type but is aberrant (mutation-type) in a minority of cases. More than 80% of undifferentiated carcinomas display evidence of epithelial differentiation in the form of intense EMA and cytokeratin (especially cytokeratin 18) staining of a small proportion of tumor cells (Fig. 10A); diffuse expression of EMA and cytokeratins is not typically found but can occur. Tumor cells typically express vimentin and a substantial number express CD138. E-cadherin labeling is absent or minimal 121,130–132. CD34 expression, which is otherwise very uncommon in epithelial neoplasms, is sometimes seen 133. Chromogranin and/or synaptophysin staining can be present, but only in a minority of tumor cells (<10%) 130. Loss of BRG-1 (the protein product of *SMARCA4*) expression can be seen (Fig. 10B), particularly in examples that have DNA MMR protein deficiency and some cases show loss of INI-1 (the protein product of *SMARCB1*) or ARID1A 125,134. BRG-1, INI-1, and ARID1A are involved in chromatin remodeling through SWI/SNF complexes 125,134. Loss of expression of MLH1 and PMS2, mostly due to *hMLH1* promoter methylation is seen in ∼50% to 60% of cases. Rare cases with germline DNA MMR gene mutations diagnostic of Lynch syndrome have been reported 135. Occasional cases may also harbor a hotspot *POLE* mutation affecting the exonuclease domain. Possible mechanisms underlying the transition from differentiated to undifferentiated carcinoma include the acquisition of mutations in *SMARCA4*, *ARID1B*, *CTNNB1*, *PPP2R1A* or *TP53* 136.

### Differential Diagnosis

Accurate diagnosis is important in view of the poor prognosis of undifferentiated/dedifferentiated carcinoma, and this is likely to become even more important as tumor subtype-specific targeted therapies are developed.

The differential diagnosis of undifferentiated carcinoma includes grade 3 endometrioid carcinoma, serous carcinoma with a solid architecture, lymphoma, plasmacytoma, melanoma, high-grade endometrial stromal sarcoma, high-grade neuroendocrine carcinoma (small cell and large cell neuroendocrine carcinoma), various metastatic carcinomas, rhabdomyosarcoma, undifferentiated sarcoma and other sarcomas, all of which can be identified with careful morphologic evaluation and the prudent use of immunohistochemical stains. As well as these neoplasms, dedifferentiated carcinoma may be confused with grade 2 endometrioid carcinoma and carcinosarcoma.

Many pleomorphic epithelial tumors were likely diagnosed as “undifferentiated carcinoma” before the recognition of the monomorphic tumors currently categorized as undifferentiated carcinomas. Anecdotal experience suggests that many, if not most, pleomorphic carcinomas are merely an extreme end of the spectrum of high-grade endometrial carcinomas, such as serous carcinomas. As with other pleomorphic tumors, the differential diagnosis with melanoma, sarcoma, and hematolymphoid tumors may need to be explored and immunohistochemistry will assist in this distinction.

In contrast to grade 3 endometrioid carcinomas, undifferentiated carcinomas are dyscohesive and do not exhibit epithelial formations, such as glands, although occasional nests and trabeculae are allowed. Undifferentiated carcinoma forms diffuse monotonous sheets, sometimes in a myxoid stroma. Most grade 3 endometrioid carcinomas, on the other hand, are composed of cohesive cells with at least focal glandular formation and squamous differentiation, which may be abortive. While gland formation excludes a diagnosis of undifferentiated carcinoma, the distinction of “true” glands from lacunar-type spaces occurring secondary to apoptosis/necrosis or artifact can be problematic and subjective. The distinction between undifferentiated carcinoma and grade 3 endometrioid carcinoma may be particularly problematic in poorly fixed specimens and sometimes cannot be resolved, even with immunohistochemistry; such cases are probably best diagnosed as “high-grade endometrial carcinoma” with an explanatory note.

Dedifferentiated carcinomas with a significant low-grade glandular component may be misclassified as grade 2 endometrioid carcinoma, significantly underestimating their aggressive behavior 116,124. Dedifferentiated carcinomas may also be confused with grade 3 endometrioid carcinomas but the former is a more overtly “biphasic” neoplasm in view of its distinct low-grade endometrioid and undifferentiated components. However, extensive sampling may be required to identify the differentiated component of dedifferentiated carcinoma. Although by definition grade 3 endometrioid carcinoma shows a predominant solid growth pattern, up to 49% of the tumor can show glandular differentiation and there is often an intimate admixture of the solid and glandular components within the same nests. The cells within the solid areas and those forming glands usually have similar cytologic appearances. In contrast, the glandular and diffuse elements in dedifferentiated carcinoma are typically separate, and the diffuse component shows greater cytologic atypia than the low-grade glandular component. A solid cohesive growth pattern is characteristic of grade 3 endometrioid carcinomas whereas cellular dyscohesion and rhabdoid morphology are more characteristic of the undifferentiated component of dedifferentiated carcinoma. The distinction between dedifferentiated carcinoma and carcinosarcoma is discussed in the section on carcinosarcoma.

Immunohistochemically, undifferentiated carcinomas and the undifferentiated component of dedifferentiated carcinomas often show loss of PAX8, E-cadherin, ER, and PR immunoreactivity 121,130. In contrast, grade 3 endometrioid carcinomas usually retain PAX8 expression while hormone receptor expression is variable 8. Loss of BRG1 and/or INI1 expression has been reported in undifferentiated carcinoma and the undifferentiated component of dedifferentiated carcinomas 125,134; loss of another subunit of the SWI/SNF complex, BAF250a (the protein product of *ARID1A*) is common in both low-grade and high-grade endometrial carcinomas 137,138. Overall, therefore a cytokeratin/EMA-focal and PAX8/ER/E-cadherin-negative immunoprofile, along with loss of BRG-1 or INI-1 expression would favor undifferentiated or dedifferentiated carcinoma over grade 3 endometrioid carcinoma (Box 3).

**BOX 3:**
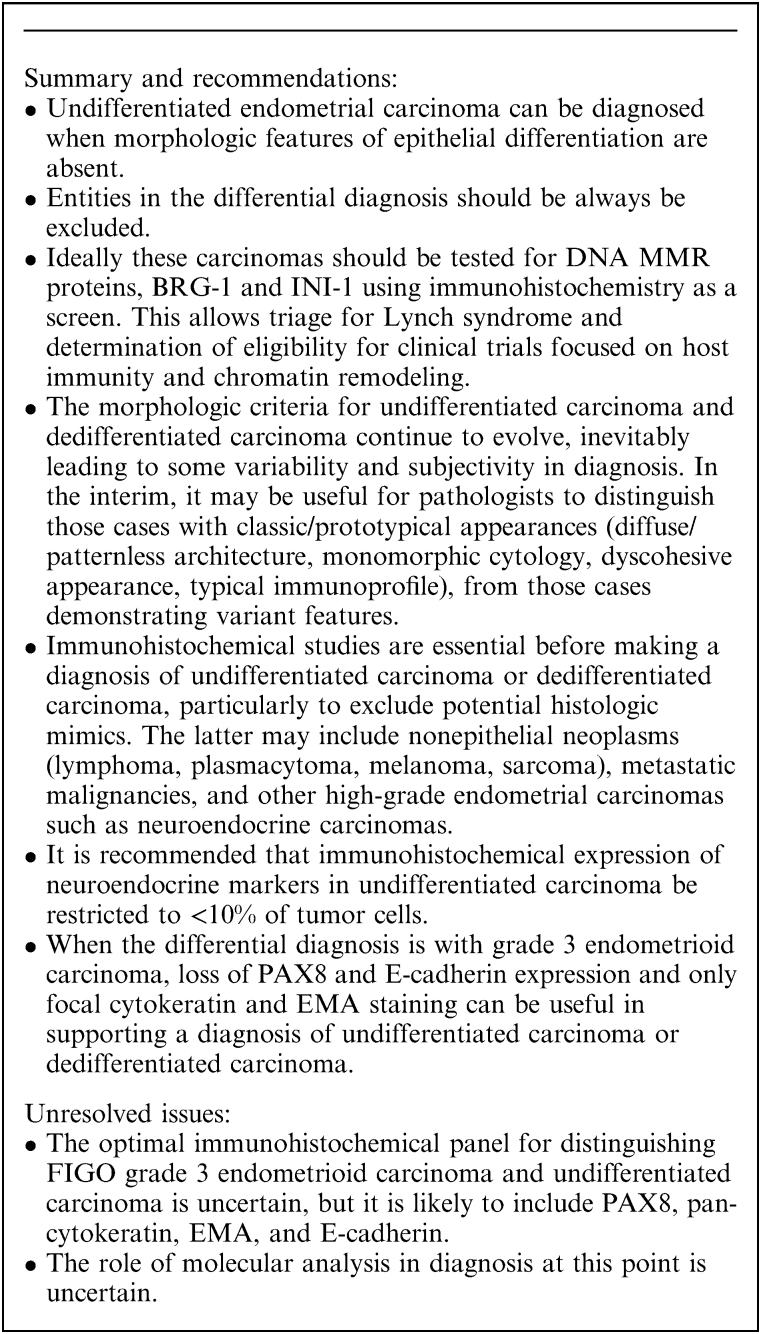
Undifferentiated and Dedifferentiated Carcinoma

## CARCINOSARCOMA

### Definition

Carcinosarcoma (formerly termed “malignant mixed Müllerian tumor”) is a biphasic endometrial carcinoma composed of a component of high-grade sarcoma (with or without heterologous elements) juxtaposed with a high-grade carcinoma of the following types: high-grade endometrioid carcinoma, serous carcinoma, clear cell carcinoma, undifferentiated carcinoma, or a histologically ambiguous, high-grade adenocarcinoma. The mesenchymal (sarcomatous) component is presumed to derive from the epithelial component (or concurrent with it from the same progenitor cell) via shifts in differentiation.

### Key Morphologic Features

By definition, carcinosarcoma consists of a high-grade carcinomatous component admixed with malignant mesenchymal elements. There is often a sharp demarcation between the carcinomatous and sarcomatous elements. While immunohistochemistry generally plays a limited role in diagnosis, broad-spectrum cytokeratins may be helpful in problematic cases, by distinguishing between poorly differentiated carcinomatous and sarcomatous elements. Specific skeletal muscle markers (especially myogenin) may be useful in confirming heterologous rhabdomyoblastic differentiation.

The presence of even minimal amounts of carcinomatous epithelium has traditionally been used to qualify a predominantly sarcomatous tumor as a carcinosarcoma 139. The literature is inconsistent on the volume of sarcomatous differentiation required to label a tumor as a carcinosarcoma, which has ranged from as little as 2% 140 to 25% 141. The authors recommend that the sarcomatous component should measure at least 1 mm in one dimension; while this is an arbitrary figure that is not evidence-based, it reflects the authors’ view that a minimal sarcomatous component should not result in a diagnosis of carcinosarcoma. The clinical significance, if any, of minimal volume sarcoma in a predominantly carcinomatous tumor has not been systematically studied. In contrast, high volume sarcomatous differentiation may portend more aggressive behavior and poor prognosis 139,140,142–144. Some studies also suggest that such largely sarcomatous tumors tend to be associated with pure sarcomatous metastases, which spread preferentially via lymphohematogenous routes to lymph nodes and distant sites, without peritoneal spread 139,140,142,143. This is in contrast to most carcinosarcomas in which the epithelial component preferentially metastasizes to peritoneal sites.

**FIG. 11:**
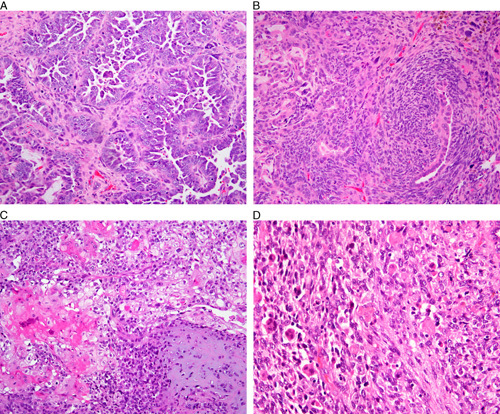
Carcinosarcoma. (A and B) Examples of homologous carcinosarcoma. (C and D) Examples of carcinosarcoma with heterologous elements, with cartilaginous differentiation (C) and rhabdomyoblastic differentiation (D).

Carcinosarcomas are divided into homologous and heterologous types, according to whether the mesenchymal component exhibits differentiation that is intrinsic (endometrial stromal sarcoma or leiomyosarcoma; Figs. 11A, B) or extrinsic (chondrosarcoma, Fig. 11C; rhabdomyosarcoma, Fig. 11D; etc.) to the uterus. Accurate subclassification of the sarcomatous component is a useful exercise but is not currently relevant to management. This separation was based on reports suggesting a more ominous prognosis for heterologous tumors 145,146 but this remains controversial. One follow-up study concluded that the prognosis for both homologous and heterologous carcinosarcomas was equally poor 139. However, a more recent report found significantly poorer 3-yr survival in patients with stage I heterologous tumors (45%) than in those with homologous tumors (93%) 147. The authors concluded that heterologous carcinosarcomas exhibited true sarcomatous differentiation and that homologous tumors were best classified as metaplastic carcinomas based on their behavioral similarity to high-grade endometrial carcinomas.

The morphology of metastases from carcinosarcomas is variable but the majority contain an epithelial component. One study 142 evaluated the cellular composition of 62 metastases, 51 of which were diagnosed at the time of surgery. Carcinoma or carcinosarcoma accounted for over 90% of metastatic tumors, with only a few comprising pure sarcoma. The characteristics of the stromal component, including grade, mitotic index, and the presence and types of heterologous elements, has not been associated with outcome in most studies. In contrast, an epithelial component consisting of serous carcinoma correlates with a higher frequency of metastases. Features associated with poor outcome in carcinomas, such as deep myometrial invasion, lymphovascular space invasion, and cervical involvement, are also associated with adverse prognosis in carcinosarcomas 139,142.

A recent large-scale molecular genetic analysis of uterine carcinosarcomas by the Cancer Genome Atlas 148 revealed extensive copy-number alterations and highly recurrent somatic mutations, most frequently in *TP53*, *PTEN*, *PIK3CA*, *PPP2R1A*, *FBXW7*, and *KRAS*. The similarity in mutational profile to uterine endometrioid and serous carcinomas provides genomic support for the categorization of carcinosarcomas as a subset of uterine carcinomas. A proportion of carcinosarcomas was also characterized by an epithelial-to-mesenchymal transition (EMT) gene signature 148.

In practice, tumors with malignant epithelial and mesenchymal differentiation are reported as carcinosarcomas, with additional comments clarifying the cell type and grade of the epithelial component (although typing of the epithelial component may be particularly difficult in carcinosarcomas), and other parameters that are associated with prognosis in epithelial tumors. The presence of heterologous elements and their differentiation should also be noted.

### Differential Diagnosis of Carcinosarcoma

A number of neoplasms may enter into the differential diagnosis of carcinosarcoma (Box 4).

**BOX 4:**
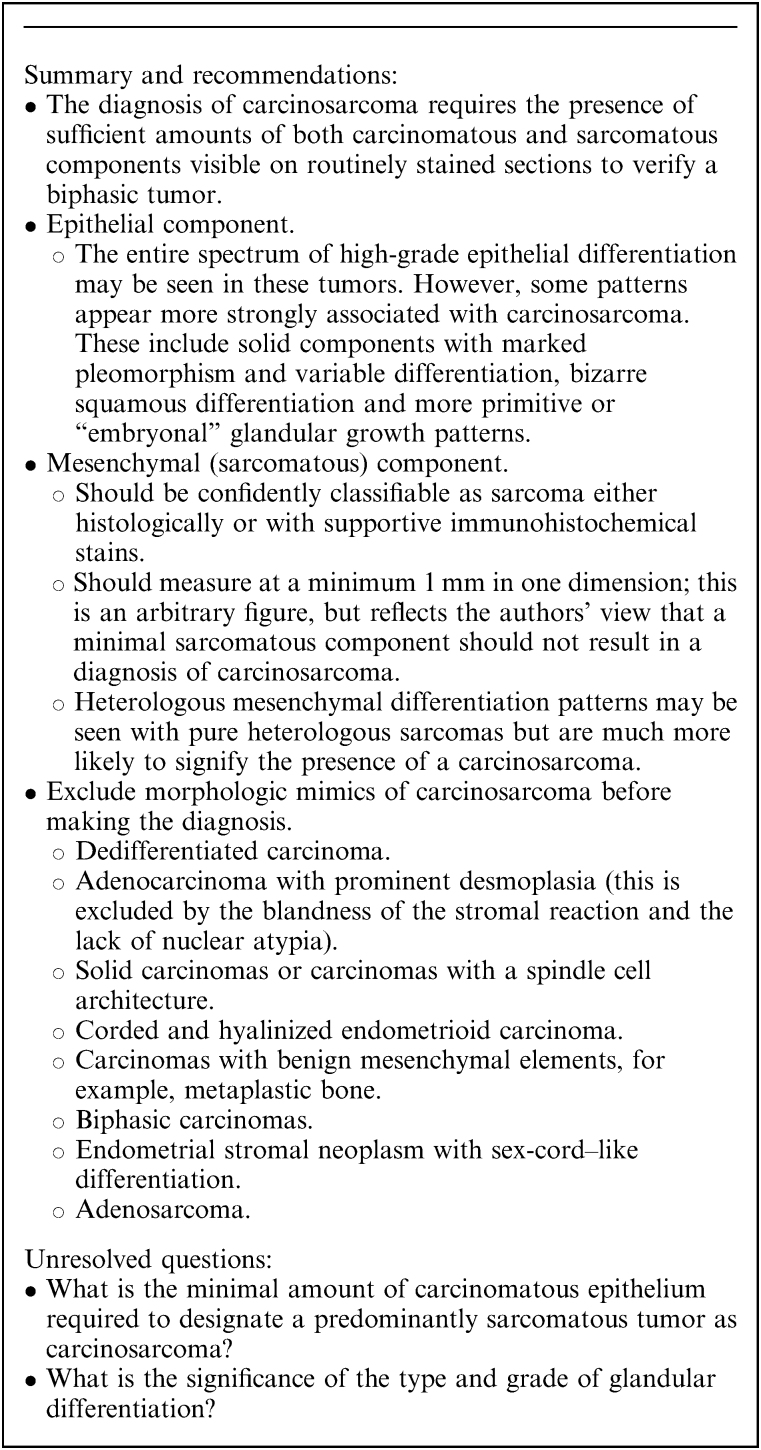
Undifferentiated and Dedifferentiated Carcinoma

Dedifferentiated carcinomas and carcinosarcomas show a low-power biphasic appearance but in the case of carcinosarcoma, the more diffuse tumor component comprises cytologically malignant mesenchymal elements that are typically spindled and pleomorphic and may exhibit heterologous differentiation 149. This contrasts with the monotonous round cells of the undifferentiated component of most dedifferentiated carcinomas. While dedifferentiated carcinoma can rarely contain a population of somewhat spindled cells, this is usually a minor feature in contrast to the more overtly sarcomatous appearances of carcinosarcoma. Many carcinosarcomas exhibit heterologous mesenchymal differentiation, most commonly in the form of chondrosarcoma, rhadomyosarcoma or osteosarcoma, but this is not a feature of dedifferentiated carcinoma. The epithelial elements of these tumors also differ in that the glandular component of dedifferentiated carcinoma is most often a low-grade endometrioid carcinoma, whereas in most carcinosarcomas, the epithelial element is a high-grade carcinoma (serous, clear cell, grade 3 endometrioid carcinoma or difficult-to-classify high-grade adenocarcinoma). However, the carcinomatous component of a carcinosarcoma can have undifferentiated appearances, at least in some areas, and the distinction from undifferentiated carcinoma or dedifferentiated carcinoma may be very difficult, particularly in biopsy specimens.

Immunohistochemistry has limited utility in this differential diagnosis. Typically, cytokeratins are diffusely expressed by the epithelial component of carcinosarcomas and there is sometimes focal staining of the mesenchymal elements 150. The latter may also express more specific mesenchymal markers if heterologous differentiation is present (eg, myogenin in rhabdomyosarcoma). Vimentin and p16 staining are of limited value since many dedifferentiated carcinomas are positive 130,132. Similarly, PAX8 is not useful in this differential diagnosis since the mesenchymal component of carcinosarcoma, like the undifferentiated component of dedifferentiated carcinoma, is usually PAX8-negative 151. As noted above, loss of MMR protein expression is relatively common (∼50%–60% of cases) in dedifferentiated carcinoma 118,125,130, and some tumors arise in patients with Lynch syndrome 152. In contrast, abnormal MMR protein expression is much less common in carcinosarcoma; the Cancer Genome Atlas analysis found microsatellite instability in 2 of 57 (4%) cases 148, although other studies have reported frequencies of MMR-deficient cases ranging between 6% and 33% 153,154.
